# A Novel 5-Methylcytosine- and Immune-Related Prognostic Signature Is a Potential Marker of Idiopathic Pulmonary Fibrosis

**DOI:** 10.1155/2022/1685384

**Published:** 2022-10-08

**Authors:** Tao Huang, Hua-Fu Zhou

**Affiliations:** ^1^The Affiliated Hospital of Youjiang Medical University for Nationalities, Department of Cardiothoracic Vascular Surgery, Baise, Guangxi Zhuang Autonomous Region, China; ^2^The First Affiliated Hospital of Guangxi Medical University, Department of Cardiothoracic Surgery, Nanning, Guangxi Zhuang Autonomous Region, China

## Abstract

Idiopathic pulmonary fibrosis (IPF) is the most common and highly lethal pulmonary interstitial lung disease. The current study is aimed at investigating reliable markers suitable for the treatment and identification of IPF. This study constructed the first 5-methylcytosine- (m5C-) and immune-related prognostic signature (m5CPS) based on coexpressed genes of m5C regulatory genes and immune-related genes. The m5CPS was established using the training cohort (*n* = 68) and verified using the test (*n* = 44) and validation (*n* = 64) cohorts. The area under the curve (AUC) values were utilized to evaluate the accuracy of m5CPS in predicting the survival of IPF patients. The Kaplan-Meier curves and Cox regression analyses were used to assess the prognostic effect of m5CPS. The AUC was utilized to evaluate the reliability of m5CPS in distinguishing IPF patients from healthy individuals. In terms of the results, m5CPS could predict the one-, three-, and five-year survival rates of IPF patients with high accuracy (AUC = .803–.973). In fact, m5CPS is not only an independent indicator of the poor prognosis of IPF patients (hazard ratio > 1; *p* < .05) but can also distinguish IPF patients from healthy individuals (AUC = .862). Also, m5CPS may affect the immune response and inflammatory response, and it was positively associated with the infiltration levels of active mast cells (*p* < .05). In sum, the current study establishes a novel m5CPS for IPF and reveals the role of m5CPS as a reliable marker for predicting the prognosis and disease status of IPF patients.

## 1. Introduction

Idiopathic pulmonary fibrosis (IPF) ranks first in incidence in terms of interstitial lung diseases, given the fact that there are 5–20 persons per 100,000 suffering from this disease in the United States and Europe annually [1, 2]. Pirfenidone and nintedanib, which are approved by the Food and Drug Administration, are used for the clinical treatment of IPF because they benefit certain IPF patients by delaying lung function decline. However, the tolerability of the two drugs poses difficulty for clinicians; worse still, they are not considered a cure for IPF. Rather, lung transplantation is the only way to cure IPF; nevertheless, problems concerning organ sources and techniques make it significantly difficult to carry out this treatment [3]. Thus, the predicted five-year survival probability of IPF patients is about 40%, and most IPF patients die two to three years after diagnosis [4–6]. Unfortunately, little is known about the efficient markers of IPF in terms of identifying the prognosis and disease status. Thus, this issue still requires further investigation.

Ribonucleic acid (RNA) methylations are a series of modifications constantly occurring in the epigenetics of eukaryotes, affecting essential genes in the progression of multiple diseases. Based on analyzing the RNA methylation-related regulatory genes, a series of prognostic signatures were constructed and considered reliable markers for a variety of diseases in previous studies. For instance, a signature developed based on N6-methyladenosine regulatory genes was identified as a prognostic risk marker for head and neck squamous cell carcinoma, and the signature may be related to the level of immune infiltration [7]. Another signature consisted of 11 N1-methyladenosine regulatory genes, which also demonstrated a close association with the prognosis of hepatocellular carcinoma and may affect the progression of the disease [8]. Conclusively, the application of RNA methylations in prognostic models may improve the understanding of diseases and provide effective prognostic indicators. Nevertheless, most of the present studies on RNA methylation modifications are related to cancers, and some regulatory mechanisms of RNA methylation have been rarely investigated, such as 5-methylcytosine (m5C) and 7-methylguanosine. For IPF, no studies on m5C exist. Thus, the research on m5C may provide novel approaches to the diagnosis and treatment of IPF.

In this study, for the first time, a reliable prognostic signature that can be used to predict and identify IPF patients was constructed based on m5C's regulatory-related genes (m5CRGs) and immune-related genes (IRGs). The m5C- and immune-related prognostic signature (m5CPS) was established using a training cohort and validated using another two cohorts. Moreover, corresponding analyses were also performed to promote the understanding of m5C in IPF, including prospective information about the clinical application potential, underlying molecular functions and signaling pathways, and underlying drugs regarding m5CPS in IPF.

## 2. Materials and Methods

### 2.1. Patient and Clinical Parameter Data

Cell samples from the bronchoalveolar lavage (BAL) of 176 IPF patients and 20 healthy control persons used in this study were collected from the Gene Expression Omnibus database with series number GSE70866, which contained two cohorts—GPL14550 and GPL17077. At the same time, the clinical parameters (including GAP (gender-age-physiologic) scores, age, and gender) of the patients in the two cohorts were also obtained. A log_2_ (*x* + 1) algorithm was applied to normalize the gene expression profile of both the GPL14550 and GPL17077 cohorts.

### 2.2. Development and Validation of m5CPS

Ten m5CRGs—*DNMT3A* (DNA methyltransferase 3 alpha), *DNMT3B* (DNA methyltransferase 3 beta), *NOP2* (NOP2 nucleolar protein), *NSUN* (NOP2/Sun RNA methyltransferase) 2–7, and *TET2* (tet methylcytosine dioxygenase 2)—that were obtained from the previous literature [9] and included in the GPL14550 and GPL17077 cohorts were analyzed in this study. A Spearman correlation analysis was applied to select m5CRGs coexpressed genes (m5C-CEGs) based on the absolute value of *ρ* > .5 (*p* < .05). The univariate Cox regression analysis was utilized to identify prognosis-related genes (PRGs) of IPF. IRGs (*n* = 1,793) were obtained from the ImmPort Portal on March 7, 2022. Candidate genes for the development of m5CPS were selected in the intersection of m5C-CEGs, PRGs, and IRGs.

The “sample” algorithm can be used to divide a cohort into several cohorts randomly, based on which some IPF patients from the GPL14550 dataset were identified as the training cohort (*n* of IPF = 68), and the remainder of IPF individuals were set as the test cohort (*n* of IPF = 44). GPL17077 was considered an independent validation cohort (*n* of samples = 64).

The least absolute shrinkage and selection operator (LASSO) Cox regression was applied to develop m5CPS for the training cohort (including 68 IPF patients). The area under the curve (AUC) values of time-dependent receiver operating characteristic curves [10] were utilized to evaluate the accuracy of m5CPS in predicting the prognoses of IPF patients. Both the test (including 44 IPF patients) and validation (containing 64 IPF patients) cohorts were used to verify the reliability of m5CPS in IPF.

### 2.3. The Potential Clinical Value of m5CPS in IPF

The Kaplan-Meier curves and Cox regression analyses were used to assess the prognostic effect of m5CRGs and m5CPS. A nomogram was established to determine whether m5CPS had potential in clinical application for IPF patients, which was verified by calibration curves. Decision curve analysis (DCA) [11] curves were used to determine whether there would be net benefits for IPF individuals using the nomogram. For an extensive understanding of the clinical significance of m5CPS in IPF, the AUC was utilized to evaluate the reliability of m5CPS in distinguishing IPF patients from healthy individuals via BAL cells.

### 2.4. The Underlying Molecular Functions, Signaling Pathways, and Drug Sensitivity Analyses of m5CPS in IPF

The “limma” package [12] was used to identify the differential expression genes (DEGs) between the high-risk and low-risk groups with an absolute value of log_2_(fold change) > 1 and false discovery rate < .05. These DEGs were utilized to investigate the underlying molecular functions of the gene ontology and signaling pathways of KEGG (Kyoto Encyclopedia of Genes and Genomes) [13], which were finished with the “clusterProfiler” and “GOplot” packages [14, 15].

The infiltration levels of 22 immune cells for each IPF patient in the training, test, and validation cohorts were calculated using the CIBERSORT algorithm [16]. The difference in the infiltration levels of immune cells in the high-risk and low-risk groups was assessed through the Wilcoxon rank-sum analysis. The underlying applicable drugs for IPF patients were explored by using the “oncoPredict” package [17] based on IC50 (half maximal inhibitory concentration).

### 2.5. Validation Analyses Based on Synthetic Minority Oversampling Technique Data

Given that limited healthy samples were included in the current study, more samples (120 IPF samples versus 120 healthy samples) were produced based on the GPL14550 cohort using the SMOTE (synthetic minority oversampling technique) algorithm of the “DMwR” package. The extensive samples were used to validate the different expression levels of m5CRGs between the IPF group and healthy group, the distinct expression levels of constitutive genes of m5CPS between the IPF group and healthy group, and the ability of m5CPS to distinguish IPF patients from healthy individuals.

### 2.6. Statistical Analysis

**Figure 1:**
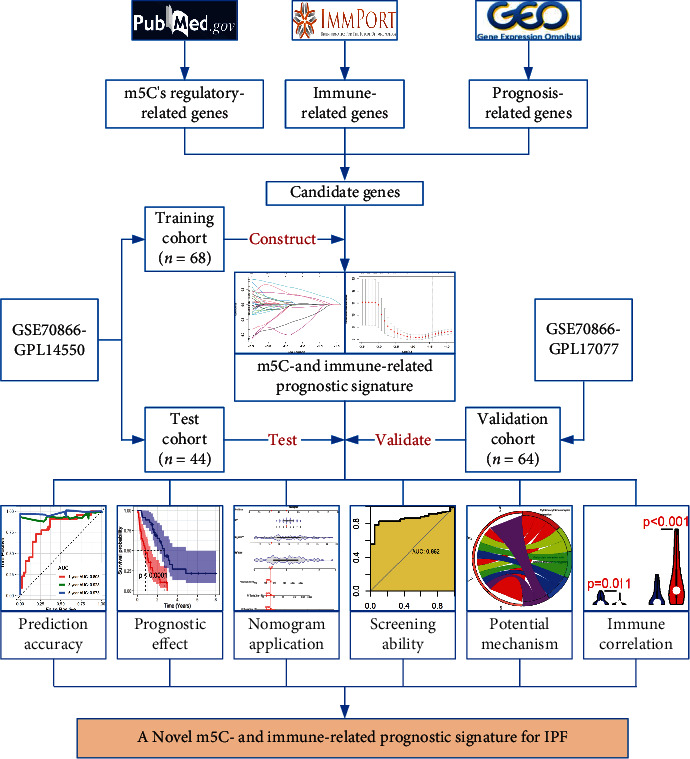
The design and main results of this study. IPF: idiopathic pulmonary fibrosis; m5C: 5-methylcytosine.

A Wilcoxon rank-sum test was applied in comparing certain risk scores between distinct groups (e.g., IPF versus control). A Spearman coefficient was utilized for the correlation analysis. All the analyses were performed in R (v4.1.0). A flow chart for this study can be referenced in Figure 1.

## 3. Results

### 3.1. The Expression Profile of m5CRGs and Their Correlation with Prognosis in IPF

**Figure 2:**
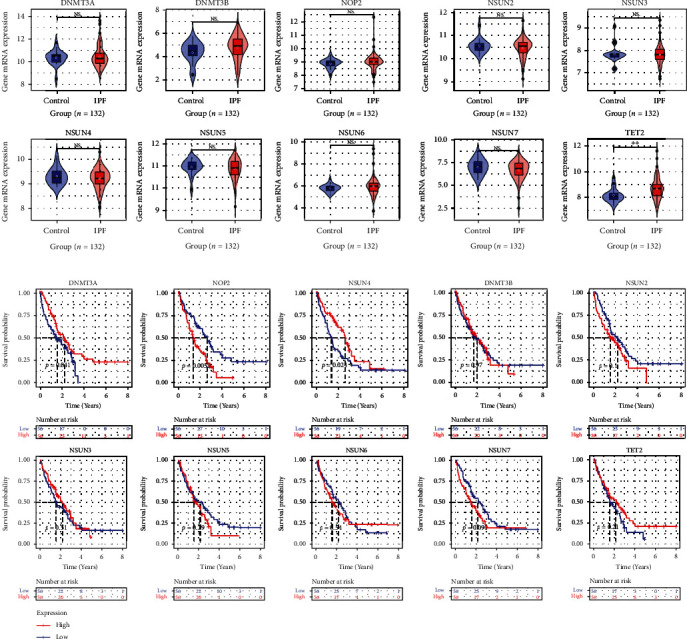
The expression and prognostic significance of m5C's regulatory-related genes (m5CRGs) in IPF. (a) Differences in expression of m5CRGs between IPF and normal bronchoalveolar lavage cells; the *p* value is calculated based on Wilcoxon rank-sum tests. ^NS^*p* > 0.05, ^∗^*p* < 0.05, ^∗∗^*p* < 0.01, and ^∗∗∗^*p* < 0.001. (b) The association between m5CRG expression levels and the prognosis in IPF patients.

Based on the GPL14550 dataset, upregulated *TET2* expression was detected in IPF rather than healthy BAL cells (*p* < .05; Figure 2(a)), and the statistical significance of the expression levels of the remaining nine m5CRGs between the IPF group and healthy group was not observed (*p* > .05; Figure 2(a)). Using the SMOTE data, increased *TET2* expression in IPF was validated, and *DNMT3B* and *NSUN6* were also found to be upregulated in the IPF group (*p* < .05; Figure S1).

Those IPF patients with the overexpression of *DNMT3A* or *NSUN4* were found to have favorable survival as compared to the rest of the patients, while IPF individuals with elevated *NOP2* were found to have poor prognoses (*p* < .05; Figure 2(b)). Moreover, the univariate Cox regression analysis supported these findings, and the multivariate Cox regression analysis identified that *DNMT3A*, *NSUN4*, and *NOP2* were independent prognostic factors for IPF patients (Table 1).

### 3.2. The Establishment of m5CPS for IPF Patients

**Figure 3:**
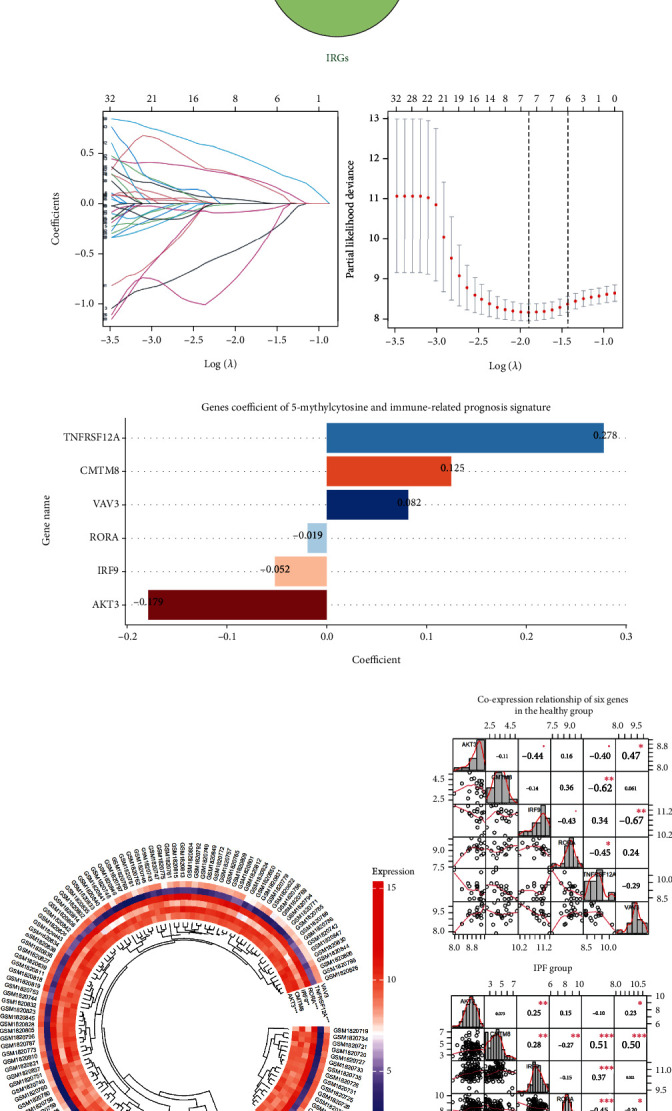
The construction of 5-methylcytosine and immune-related prognosis signature (m5CPS). (a) Sixty-three genes were identified as candidate genes for m5CPS. m5C-CEGs: m5CRGs coexpressed genes; PRGs: prognosis-related genes; IRGs: immune-related genes. (b) The least absolute shrinkage and selection operator (LASSO) coefficient profiles of the 63 candidate genes. The coefficient profile plot was produced against the log (lambda) sequence in the LASSO model. The optimal parameter (lambda) was selected. (c) Coefficients of six genes in m5CPS. (d) Differential expression levels of some of the six genes between the healthy group (the smaller fan-shaped region) and the IPF group (the larger fan-shaped region). (e) Coexpression relationship of six genes in the healthy group (the top panel) and the IPF group (the bottom panel); the Spearman correlation coefficient was used in this panel. ^∗^*p* < .05, ^∗∗^*p* < .01, ^∗∗∗^*p* < .001.

According to the GPL14550 datasets, 8,157 m5C-CEGs (*ρ* > .5 and *p* < .05) and 3,995 IPF-PRGs (*p* < .05) were selected via the correlation analysis and univariate Cox regression analysis, respectively (Figure 3(a)). Sixty-three candidate genes for developing the m5CPS were identified from the intersection of m5C-CEGs, IPF-PRGs, and IRGs (Figure 3(a)). Those IPF patients from the GPL14550 cohort were divided into the training cohort (*n* = 68, 60%) and test cohort (*n* = 44, 40%), while patients from the GPL17077 cohort were identified as the validation set for verifying the m5CPS (*n* = 64). The characteristics of the training, test, and validation cohorts can be viewed in Table 2.

Using the least absolute shrinkage and selection operator Cox regression algorithm, the m5CPS was established based on the training set (Figure 3(b)). The m5CPS consisted of six genes—*AKT3* (AKT serine/threonine kinase 3), *CMTM8* (CKLF like MARVEL transmembrane domain containing 8), *IRF9* (interferon regulatory factor 9), *RORA* (RAR related orphan receptor A), *TNFRSF12A* (TNF receptor superfamily member 12A), and *VAV3* (vav guanine nucleotide exchange factor 3), the coefficients of which are shown in Figure 3(c). Among the six genes, differential expression levels on the part of *AKT3*, *IRF9*, *RORA*, and *TNFRSF12A* were observed between the healthy group and the IPF group (*p* < .05; Figure 3(d)); this result was also supported by the SMOTE data (*p* < .05; Figure S2). The expression correlation of these six genes was not significant in the healthy group, while it was conspicuous in the IPF group (Figure 3(e)).

### 3.3. Prediction Accuracy and Prognostic Effect of m5CPS

**Figure 4:**
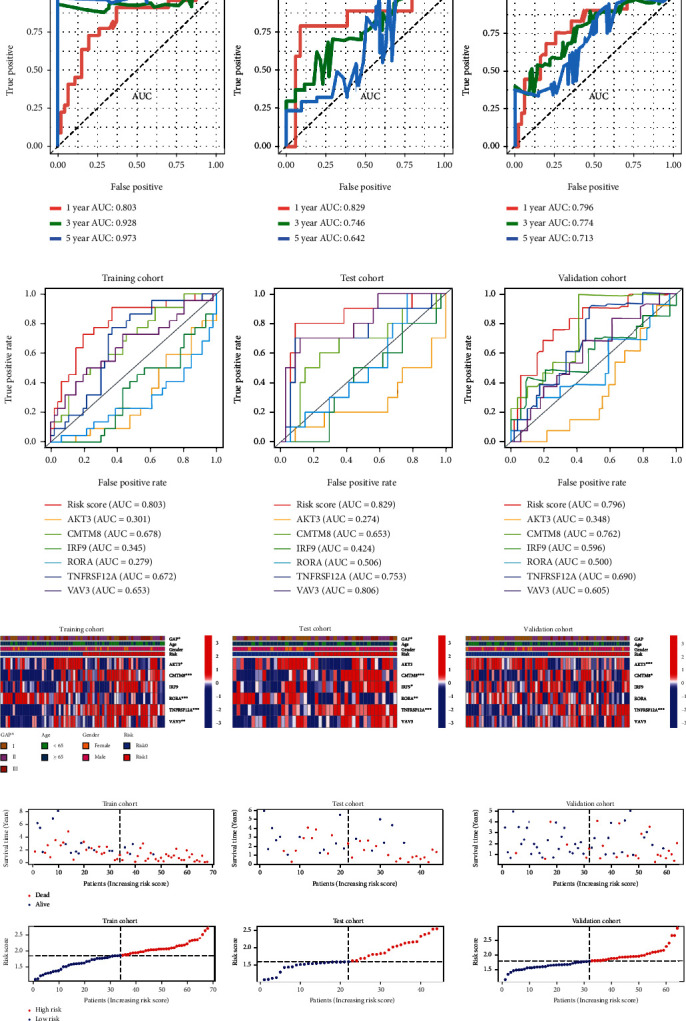
The prediction accuracy and prognostic value of m5CPS in IPF. (a) The accuracy of m5CPS in predicting the one-, three-, and five-year survival of IPF patients. (b) The accuracy of m5CPS and single genes in predicting the one-year survival of IPF patients. (c) Heatmaps of the expression profile of m5CPS and clinical characteristics in the training, test, and validation cohorts. GAP: gender-age-physiologic variables. ^∗^*p* < .05, ^∗∗^*p* < .01, and ^∗∗∗^*p* < .001. (d) Risk plots of the training, test, and validation cohorts.

Clearly, m5CPS demonstrated conspicuous accuracy in predicting both the short-term (one-year) and long-term (three-year and five-year) survival of patients from the training cohort (AUC = .803–.973), and such a phenomenon was also observed in the test and validation cohorts (AUC = .642–.829) (Figure 4(a)). Moreover, m5CPS was more excellent than any single constitutive gene of m5CPS in terms of predicting the one-year survival of IPF individuals (AUC = .796–.829; Figure 4(b)).

None of the three features—GAP, gender, or age—was found to be different between IPF patients with a high-risk score and IPF patients with a low-risk score in all of the training, test, and validation cohorts, while the difference in *CMTM8* and *TNFRSF12A* expression levels between the high-risk score group and the low-risk score group was detected (Figure 4(c)). In all of the training, test, and validation cohorts, more surviving individuals were observed in the group with a low-risk score (Figure 4(d)).

**Figure 5:**
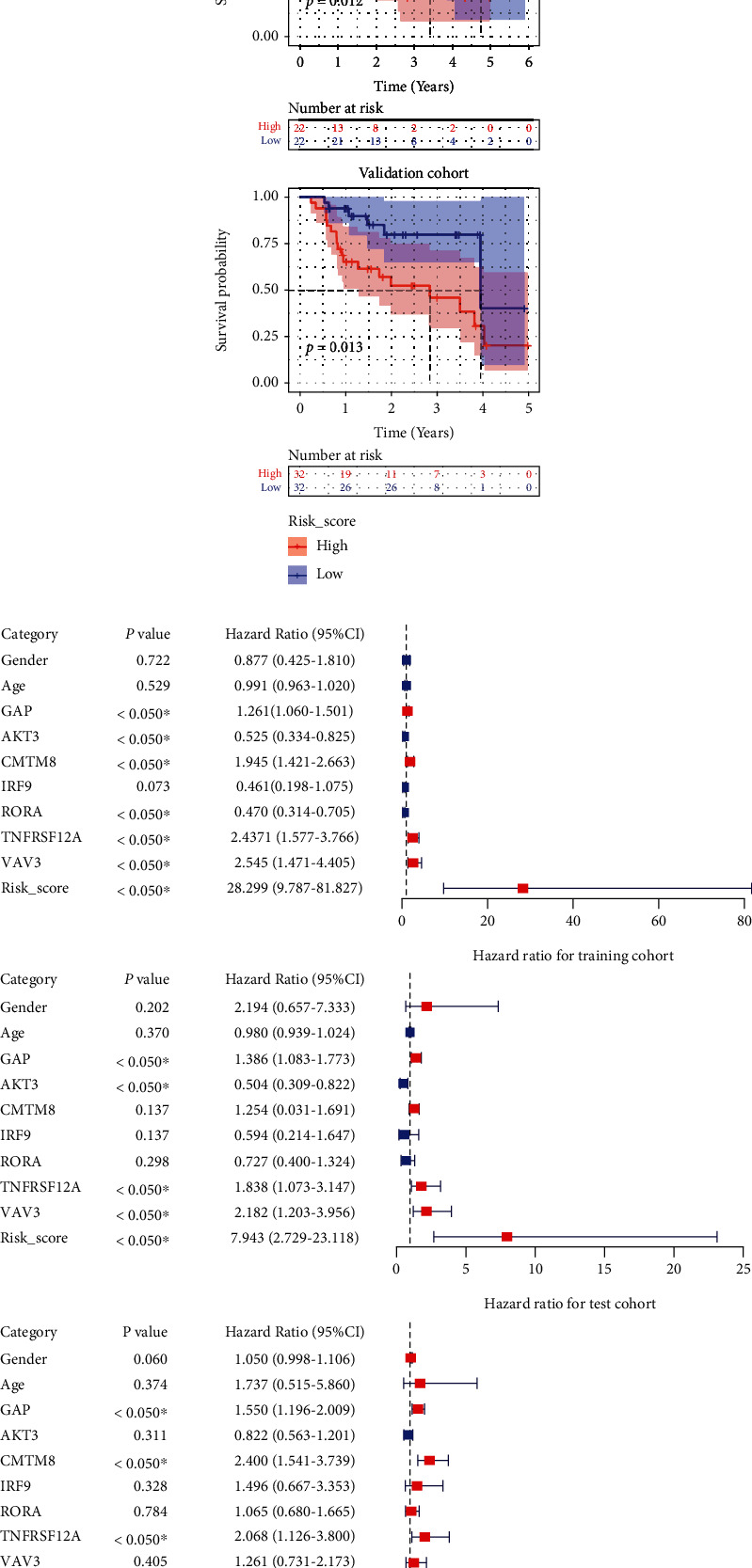
The prognostic value of m5CPS and clinical characteristics in IPF. (a) Kaplan-Meier curves. (b) Univariate Cox regression analysis. (c) Multivariate Cox regression analysis.

Based on the results of the training, test, and validation cohorts, a high m5CPS score indicated a pessimistic median survival time for IPF patients via the Kaplan-Meier curves (*p* < .05; Figure 5(a)). In the univariate Cox regression analyses, GAP, *TNFRSF12A*, and m5CPS risk scores were identified as prognostic risk factors for IPF patients in all of the training, test, and validation cohorts (hazard ratio > 1, *p* < .05; Figure 5(b)). Further multivariate Cox regression analyses confirmed the m5CPS risk score as an independent risk factor in IPF based on the three cohorts (hazard ratio > 1, *p* < .05; Figure 5(c)).

### 3.4. The Clinical Application Potential of m5CPS in IPF

**Figure 6:**
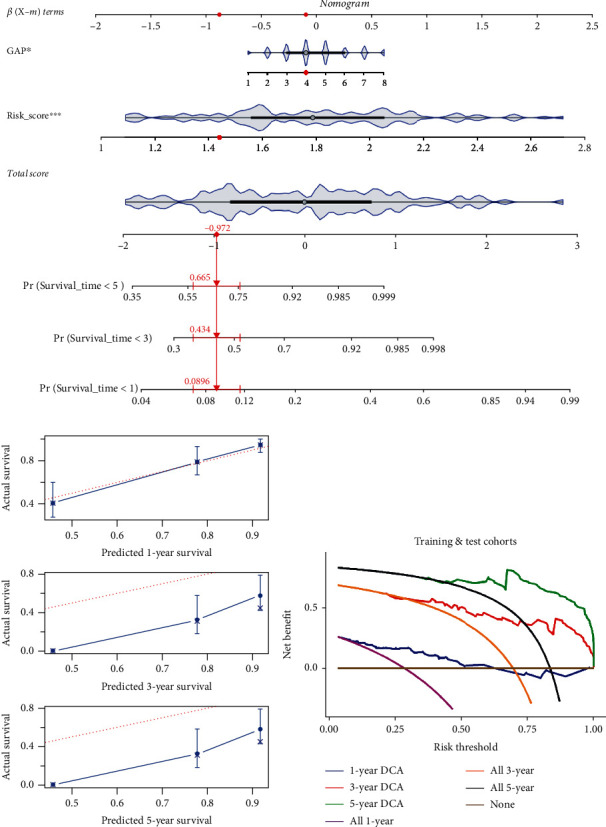
An application of the m5CPS in the nomogram. (a) The nomogram based on the GAP and m5CPS, the red dots in the nomogram represent the clinical features of patient GSM1820740. (b) Calibration curves assess the accuracy of the nomogram in predicting the survival probabilities of IPF individuals. (c) Decision curve analysis (DCA) demonstrates the positive net benefits of the nomogram for IPF patients.

The nomogram consisted of the two factors related to the prognosis of IPF in the univariate Cox regression analysis, GAP scores and m5CPS risk scores, and was constructed to explore the application potential of the signature in a clinical setting. Taking the IPF individual with the identity document “GSM1820848” as an example, the predicted probabilities (based on the nomogram) of his survival for less than one, three, and five years were 0.0896, 0.434, and 0.665, respectively (Figure 6(a)), which were calculated based on his clinical parameters—GAP score = 4 and m5CPS risk score = 1.440. By the calibration curves, although the nomogram did not show high accuracy in predicting three- and five-year survival, a reliable one-year survival rate can be predicted for IPF patients (Figure 6(b)). Moreover, it can be seen from the DCA that IPF patients will derive significant net benefits from the nomogram in terms of predicting survival probabilities (Figure 6(c)).

**Figure 7:**
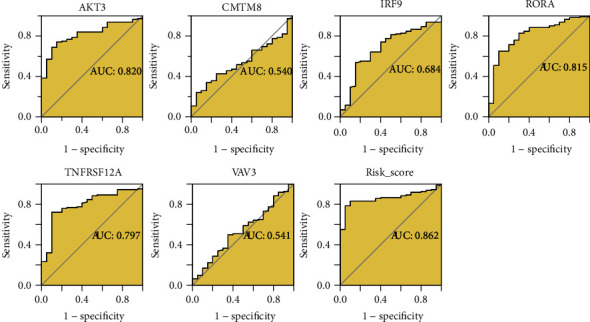
The accuracy of m5CPS and its constitutive genes in terms of distinguishing IPF bronchoalveolar lavage from healthy lung bronchoalveolar lavage. AUC: area under the curve.

Through AUC values, m5CPS and its constitutive genes in terms of distinguishing IPF patients from healthy persons were evaluated. As a result, *AKT3*, *RORA*, *TNFRSF12A*, and the m5CPS risk score made it feasible to screen IPF patients via detecting BAL cell samples (AUC ≥ .797), and among these factors, the m5CPS risk score showed the optimal effect in terms of screening for IPF patients (AUC = .862; Figure 7). Notably, the analysis results based on the SMOTE data also supported the ability of m5CPS to distinguish IPF patients from the healthy (Figure S3).

### 3.5. The Underlying Molecular Functions and Signaling Pathways of m5CPS and the Drug Sensitivity Analysis for the Signature

**Figure 8:**
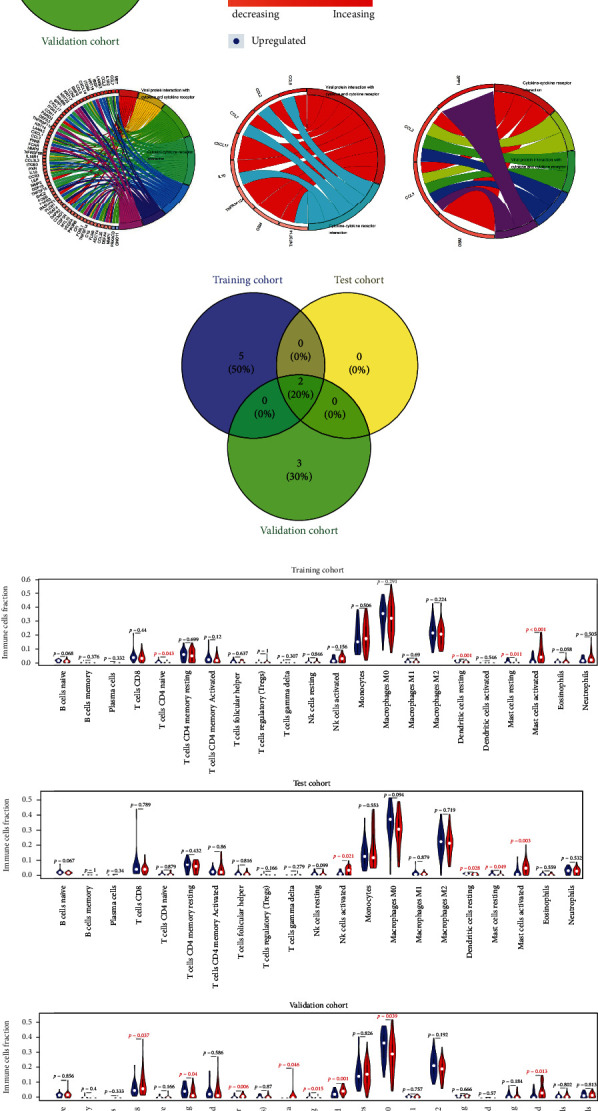
The potential molecular mechanisms of m5CPS in IPF. (a) Selection of overlap molecular functions of m5CPS in the three cohorts. (b) The overlap molecular functions of m5CPS in the three cohorts; the panel was drawn based on the validation cohort. (c) KEGG signaling pathways of m5CPS based on the three cohorts. (d) Selection of KEGG signaling pathways of m5CPS in the three cohorts. (e) The immune cell fraction levels between patients with high- and low-risk scores; *p* value was calculated based on Wilcoxon rank-sum tests.

Using gene ontology analysis based on DEGs between the high-risk and low-risk groups for all of the training, test, and validation cohorts, m5CPS may affect six types of functions, such as chemokine activity (Figures 8(a) and 8(b)). From the perspective of the KEGG signaling pathways, m5CPS was shown identified to participate in two pathways—“cytokine-cytokine receptor interaction” and “viral protein interaction with cytokine and cytokine receptor”—based on the results of the training, test, and validation cohorts (Figures 8(c) and 8(d)).

The association of m5CPS with 22 kinds of immune cells was analyzed. Ultimately, there was a positive correlation between the m5CPS risk score and the infiltration levels of active mast cells in all of three cohorts, and such a finding was also supported by the negative relationship between the m5CPS risk score and the infiltration levels of resting mast cells in both of the training and test cohorts (Figure 8(e)).

**Figure 9:**
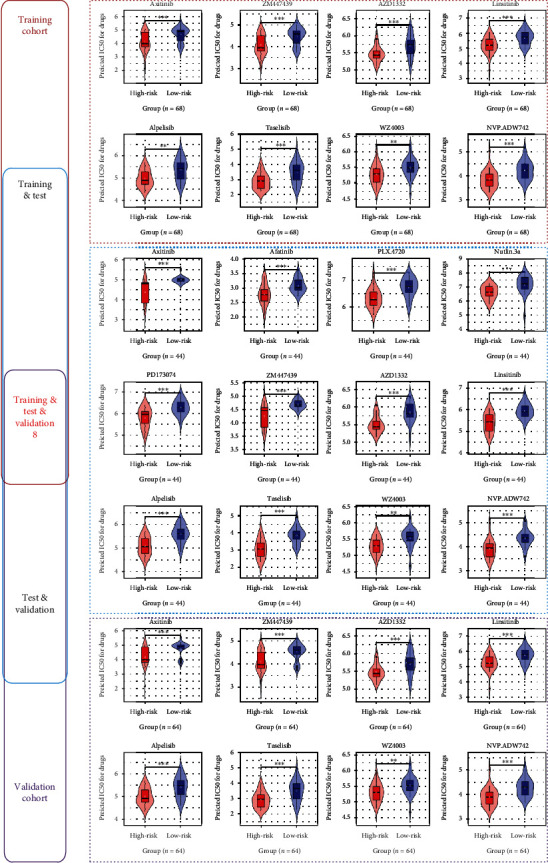
The underlying target drugs for m5CPS. *p* values were calculated based on Wilcoxon rank-sum tests. ^∗∗^*p* < 0.01 and ^∗∗∗^*p* < 0.001. The high-risk and low-risk groups were defined based on the median risk score of each cohort; for example, for patients included in the training cohort, those with not less than the median risk score of the whole cohort were assigned to the high-risk group.

At present, the effect of drug therapy on IPF is still not ideal, so it is necessary to explore drugs that may be sensitive to IPF patients. With the internal algorithm of the “oncoPredict” package based on IC50 and based on the training cohort, this study predicted that IPF patients with a high m5CPS score would be sensitive to eight drugs (*p* < .01; Figure 9). Similarly, IPF individuals with a high m5CPS score were predicted to be sensitive to twelve and eight drugs using the training and validation cohorts, respectively (*p* < .01; Figure 9). Using all the three cohorts, IPF patients with high m5CPS scores were consistently identified to be sensitive to eight drugs—axitinib, ZM447439, AZD1332, linsitinib, alpelisib, taselisib, WZ4003, and NVP.ADW742 (Figure 9).

## 4. Discussion

As mentioned above, IPF is the most common and highly lethal pulmonary interstitial lung disease. Although pirfenidone and nintedanib have been applied to certain IPF patients in clinical settings, the effectiveness of drug treatment for IPF patients is still unsatisfactory. Nothing but lung transplantation can cure IPF; however, the lack of organ sources limits the feasibility of performing this operation. Thus, more effort must be made to investigate reliable markers that may be suitable for the treatment and identification of IPF. m5C is mainly enriched in some important functional regions (e.g., the translation initiation site) of specific molecules (e.g., mRNA and long noncoding RNA) and thereby affects a series of biological functions, such as RNA stabilization and translation [18–21]. Also, IRGs can affect important biological processes, such as immune cell infiltration and immune response. Although immune imbalance and continuous inflammatory response were considered to be important links in the process of fibrosis [22, 23], few reports about them on IPF can be consulted.

Considering the potentially important role of m5CRGs and IRGs in IPF and the lack of key substances in clinical treatment of IPF, this study constructed the first m5CPS based on m5C-CEGs and IRGs and revealed the clinical value of m5CPS. In fact, m5CPS could predict the short-term and long-term survival rates of IPF patients with high accuracy. It is not only an indicator of poor prognoses for IPF patients but can also distinguish between IPF patients and healthy individuals. These potential clinical values on the part of m5CPS were identified in the training set and verified in the test and validation cohorts. Our study also revealed the potential molecular functions and signaling pathways for m5CPS regarding IPF, which may increase the understanding of the mechanism of m5C and IRGs in IPF to some extent. Finally, this study also investigated novel drugs that may apply to patients with high m5CPS scores, providing clues for drug-related research.

Previous studies have suggested the feasibility of the application of a prognostic signature in patients with IPF. For example, Li et al. [24] constructed a hypoxia- and immune-related prognostic signature and revealed that the signature was associated with the prognosis of IPF patients. However, they did not explore whether the signature had independent prognostic value (e.g., the signature was unaffected by clinical parameters such as GAP), which was observed for m5CPS in our study. Moreover, the prognosis signature constructed by Li et al. [24] was composed of nine genes, which was more complex than the m5CPS composed of six genes in our study. He et al. [25] and Li et al. [26] developed ferroptosis-related prognostic signatures for IPF. Both of these two signatures were shown to have independent prognostic value for IPF patients. However, neither He et al. [25] nor Li et al. [26] revealed whether their signatures have the potential to distinguish IPF patients from healthy individuals, and they also did not use the risk score for signatures to explore drugs that might be applicable to patients with IPF, both of which have been accomplished in our study. Thus, our study adopts the novel perspective on m5C, which may have certain novelties and advantages.

In cases of IPF, m5CPS demonstrated conspicuous clinical value. Crucially, IPF is the pulmonary interstitial disease with the worst prognosis, so it is necessary to explore effective indicators that can be directly used to predict the prognoses of IPF patients. The m5CPS constructed in our study may be such a biomarker because it has represented high accuracy in predicting one-, three-, and five-year survival in IPF patients, and the results of the training, test, and validation cohorts supported such a finding. Indeed, previous reports have revealed several prognostic markers for IPF. For example, Nakanishi et al. [27] identified the association of IL-18 binding protein with the survival of IPF patients and the gene's role as an underlying indicator of the prognosis of IPF. Sawazumi et al. [28] also found that HNF4*α* expression represented prognostic risk signaling for IPF. However, confirming the efficacy of using a single molecule as a marker may require multiqueue verification. For instance, *TNFRSF12A* was determined to be an independent risk marker for the prognosis of IPF individuals in the training cohort; nevertheless, neither the test cohort nor the validation cohort supported this finding. Instead, the m5CPS consisting of six genes was shown to be a reliable biomarker in all three cohorts. Moreover, a biomarker consisting of several molecules may have advantages in some respects. For instance, m5CPS was more excellent than any single constitutive gene of m5CPS in terms of predicting the one-year survival of IPF individuals in our study. Moreover, the nomogram consisting of GAP score and m5CPS risk score can be applied directly to effectively predict the short-time survival probabilities of IPF patients. In addition to the prognostic effect, m5CPS made it feasible to screen IPF persons from the healthy by using BAL cells with high accuracy. However, more efforts should be made to verify the screening effect of m5CPS for IPF via more convenient detection methods. For example, Aoshima et al. [29] focused on distinguishing IPF patients from non-IPF persons via serum gremlin-1, and it is more convenient for clinicians to collect serum samples than BAL cells. In sum, m5CPS may be an essential marker for evaluating the prognosis and disease status of IPF patients.

Although m5CRGs and IRGs are relevant to fibrosis [22, 23, 30], little is known about their mechanisms in IPF, which were explored in our study. A series of essential molecular functions may be linked to the role of m5CPS play in IPF. These include chemokine activity, chemokine receptor binding, cytokine activity, cytokine receptor binding, receptor-ligand activity, and signaling receptor activator activity. This makes sense because all of the constitutive genes—*AKT3* [31], *CMTM8* [32], *IRF9* [33], *RORA* [34], *TNFRSF12A* [35], and *VAV3* [36]—of m5CPS participated in the chemokines, cytokines, or signaling receptor activator activity-related molecular biological processes. This suggests that m5CPS may affect the immune response and inflammatory response, which was also supported by the two KEGG signaling pathways—“cytokine-cytokine receptor interaction” and “viral protein interaction with cytokine and cytokine receptor”—m5CRGs may take part in. In terms of the association with immune cells, Galati et al. [37] identified a decline in dendritic cells in the blood of IPF patients and the prognostic role of such for the disease. Cha et al. [38] also revealed elevated mast cells in IPF lung tissues as compared to normal lung tissues. Furthermore, among the 22 kinds of immune cells tested in our study, the m5CPS risk score was positively associated with the infiltration levels of active mast cells in all of the training, test, and validation cohorts. Thus, the previous results and our study suggested the important roles of mast cells in IPF, particularly for patients with high-risk scores. Lastly, considering that the effect of drug therapy for IPF remains poor, our study identified that IPF patients with high m5CPS scores were sensitive to eight drugs: axitinib, ZM447439, AZD1332, linsitinib, alpelisib, taselisib, WZ4003, and NVP.ADW742.

Several limitations of this study must be emphasized. Above all, we failed to collect more samples from the multicenter to verify the clinical value of m5CPS. Due to the lack of clinical parameters (e.g., pulmonary function) for IPF patients, the application of m5CPS in IPF patients is relatively limited. It is also necessary to explore and verify the potential molecular mechanism of m5CPS in vitro and in vivo in the future.

## 5. Conclusions

This study develops a novel m5CPS for IPF. The m5CPS may serve as a prediction and prognosis marker for IPF patients. This research also investigates the underlying mechanisms of IPF from the perspective of m5CRGs and IRGs, which may contribute to the understanding of IPF.

## Figures and Tables

**Table 1:** The Cox regression analyses of m5C's regulatory-related genes.

Gene	Univariate Cox regression analysis		Multivariate Cox regression analysis	
	Hazard ratio [95% CI]	*p* value	Hazard ratio [95% CI]	*p* value
*DNMT3A*	0.617 [0.387-0.983]	0.042	0.527 [0.313-0.885]	0.016
*DNMT3B*	0.992 [0.628-1.566]	0.972	—	—
*NOP2*	1.925 [1.207-3.071]	0.006	1.746 [1.068-2.857]	0.026
*NSUN2*	1.467 [0.926-2.323]	0.102	—	—
*NSUN3*	0.859 [0.545-1.355]	0.513	—	—
*NSUN4*	0.591 [0.373-0.937]	0.025	0.448 [0.273-0.734]	0.001
*NSUN5*	1.285 [0.806-2.048]	0.292	—	—
*NSUN6*	1.155 [0.733-1.820]	0.534	—	—
*NSUN7*	1.482 [0.936-2.346]	0.093	—	—
*TET2*	0.748 [0.474-1.180]	0.212	—	—

**Table 2:** The clinical characteristics of the training, test, and validation cohorts.

Clinical characteristics		Training cohort		Test cohort		Validation cohort	
		Number	Percentage	Number	Percentage	Number	Percentage
Status	Alive	18	26.5%	18	40.9%	40	62.5%
	Dead	50	73.5%	26	59.1%	24	37.5%
Gender	Female	11	16.2%	8	18.2%	13	20.3%
	Male	57	83.8%	36	81.8%	51	79.7%
Age (years)	<65	26	38.2%	11	25.0%	22	34.4%
	≥65	42	61.8%	33	75.0%	42	65.6%
GAP^a^ stages	I	15	22.1%	16	36.4%	25	39.1%
	II	33	48.5%	19	43.2%	31	48.4%
	III	20	29.4%	9	20.5%	8	12.5%

^a^Gender-age-physiologic variables.

## Data Availability

The data that support the findings of this study are available in Gene Expression Omnibus at https://www.ncbi.nlm.nih.gov/gds/.

## Supplementary Materials

Supplementary 1 Figure S1: using synthetic minority oversampling technique data, differences in expression on the part of m5CRGs between IPF and normal bronchoalveolar lavage cells; the *p* value is calculated based on the Wilcoxon rank-sum tests. ^NS^*p* > 0.05; ^∗^*p* < 0.05; ^∗∗^*p* < 0.01; ^∗∗∗^*p* < 0.001.

Supplementary 2 Figure S2: using synthetic minority oversampling technique data, differential expression levels of six genes between the healthy group (the bottom fan-shaped region) and IPF group (the top fan-shaped region).

Supplementary 3 Figure S3: using synthetic minority oversampling technique data, the accuracy of m5CPS and its constitutive genes in distinguishing IPF bronchoalveolar lavage from healthy lung bronchoalveolar lavage. AUC: area under the curve.
