# Supplementary figures and images for: A Novel 5-Methylcytosine- and Immune-Related Prognostic Signature Is a Potential Marker of Idiopathic Pulmonary Fibrosis
Source: Comput Math Methods Med. 2022 Oct 8;2022:1685384. doi: 10.1155/2022/1685384 (PMC9574547; doi:10.1155/2022/1685384)

DNMT3A

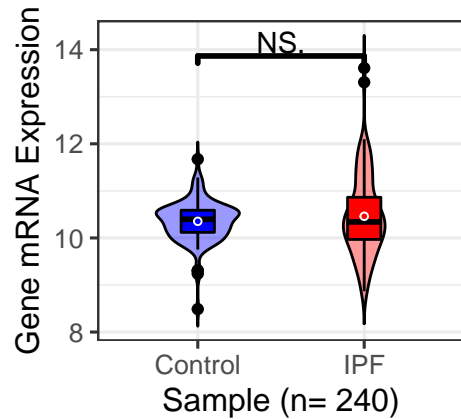

DNMT3B

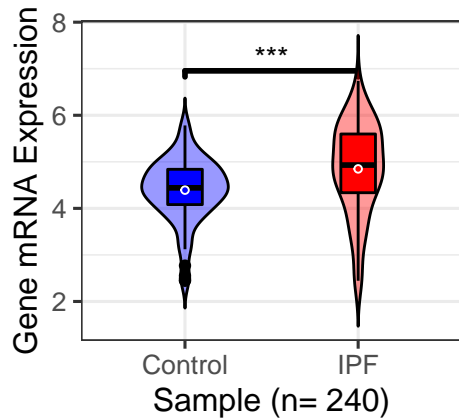

NOP2

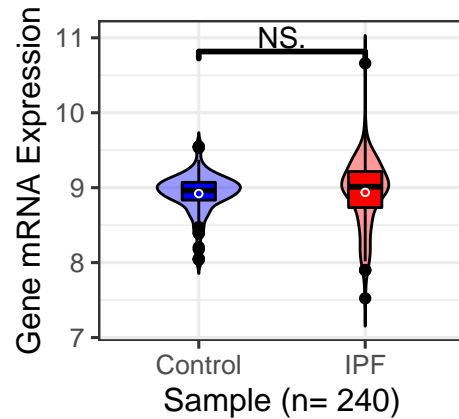

NSUN2

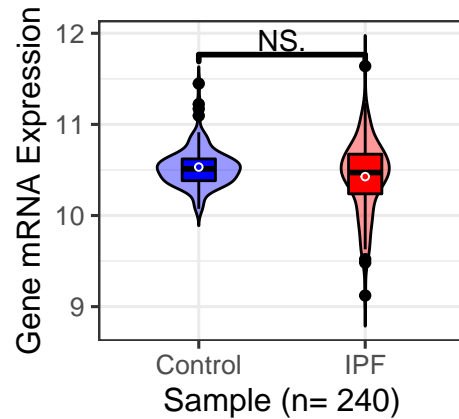

NSUN3

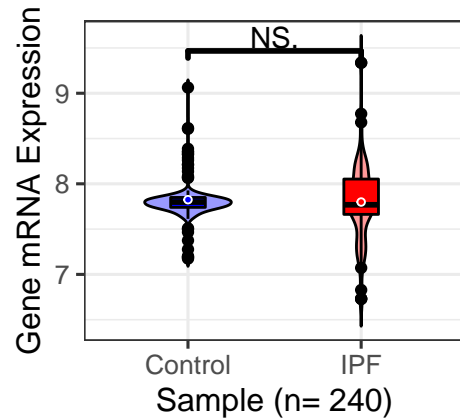

NSUN4

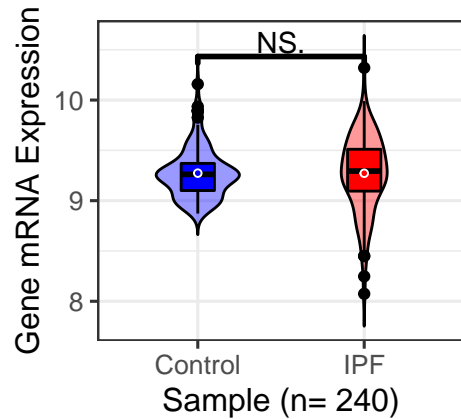

NSUN5

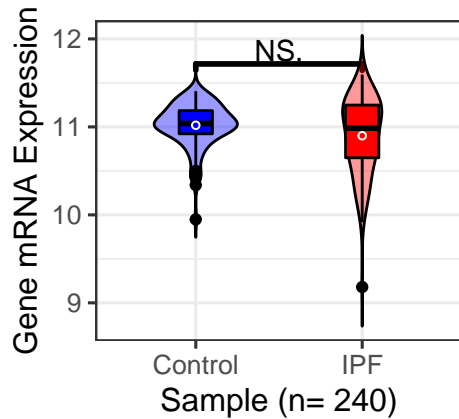

NSUN6

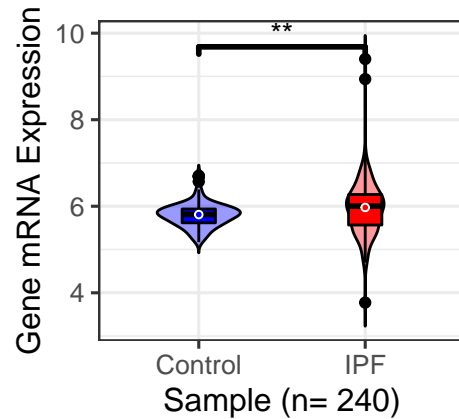

NSUN7

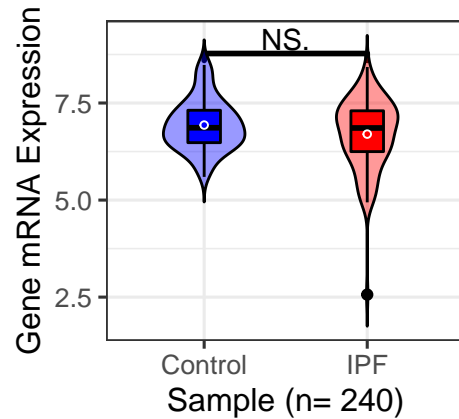

TET2

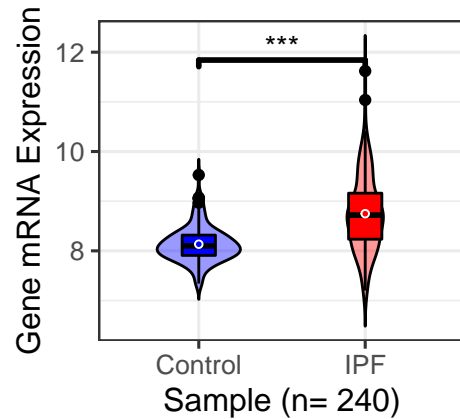

Supplement: Supplementary 1 — Figure S1: using synthetic minority oversampling technique data, differences in expression on the part of m5CRGs between IPF and normal bronchoalveolar lavage cells; the p value is calculated based on the Wilcoxon rank-sum tests. NSp > 0.05; ∗p < 0.05; ∗∗p < 0.01; ∗∗∗p < 0.001. [file 1685384.f1.pdf]

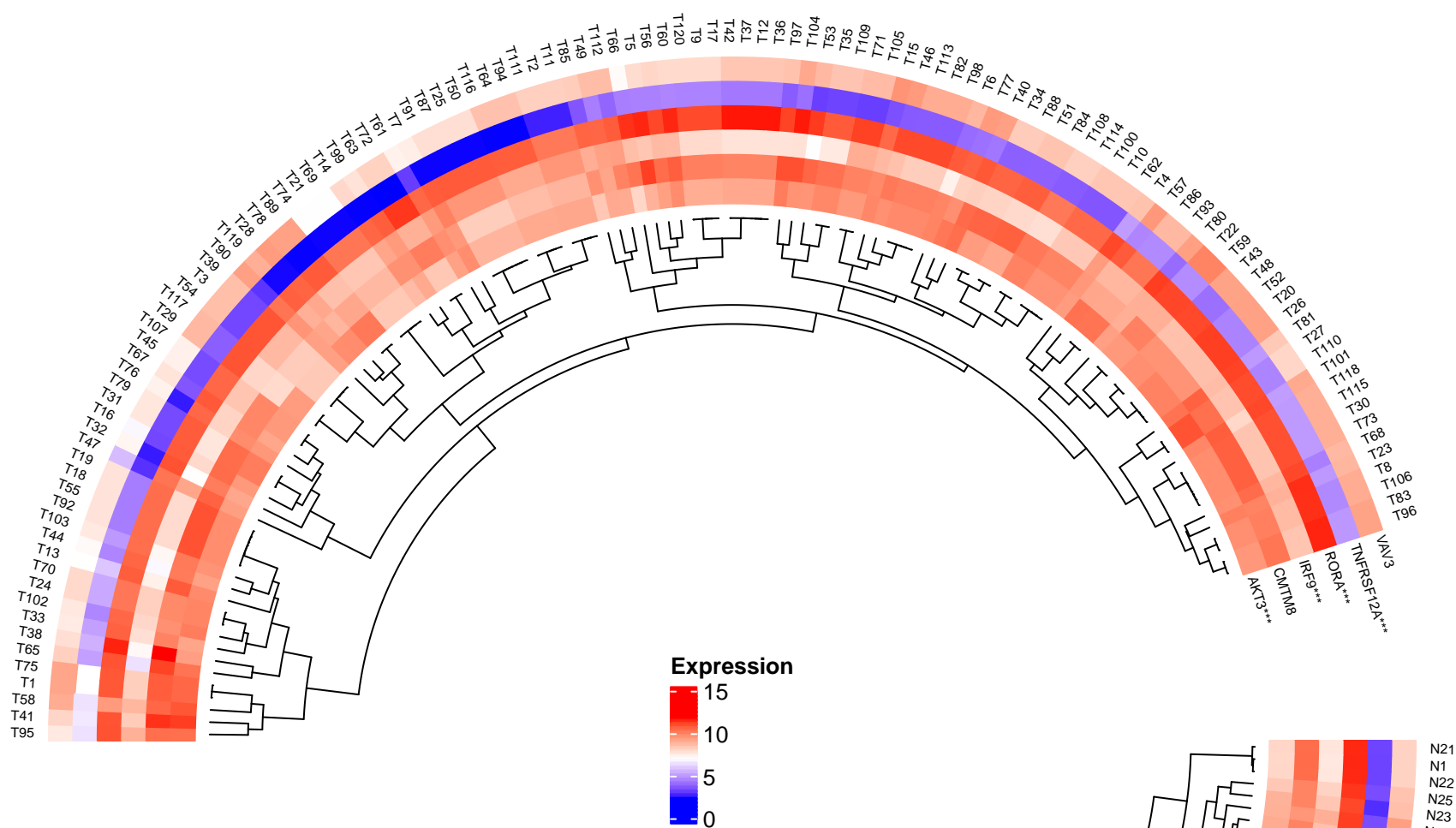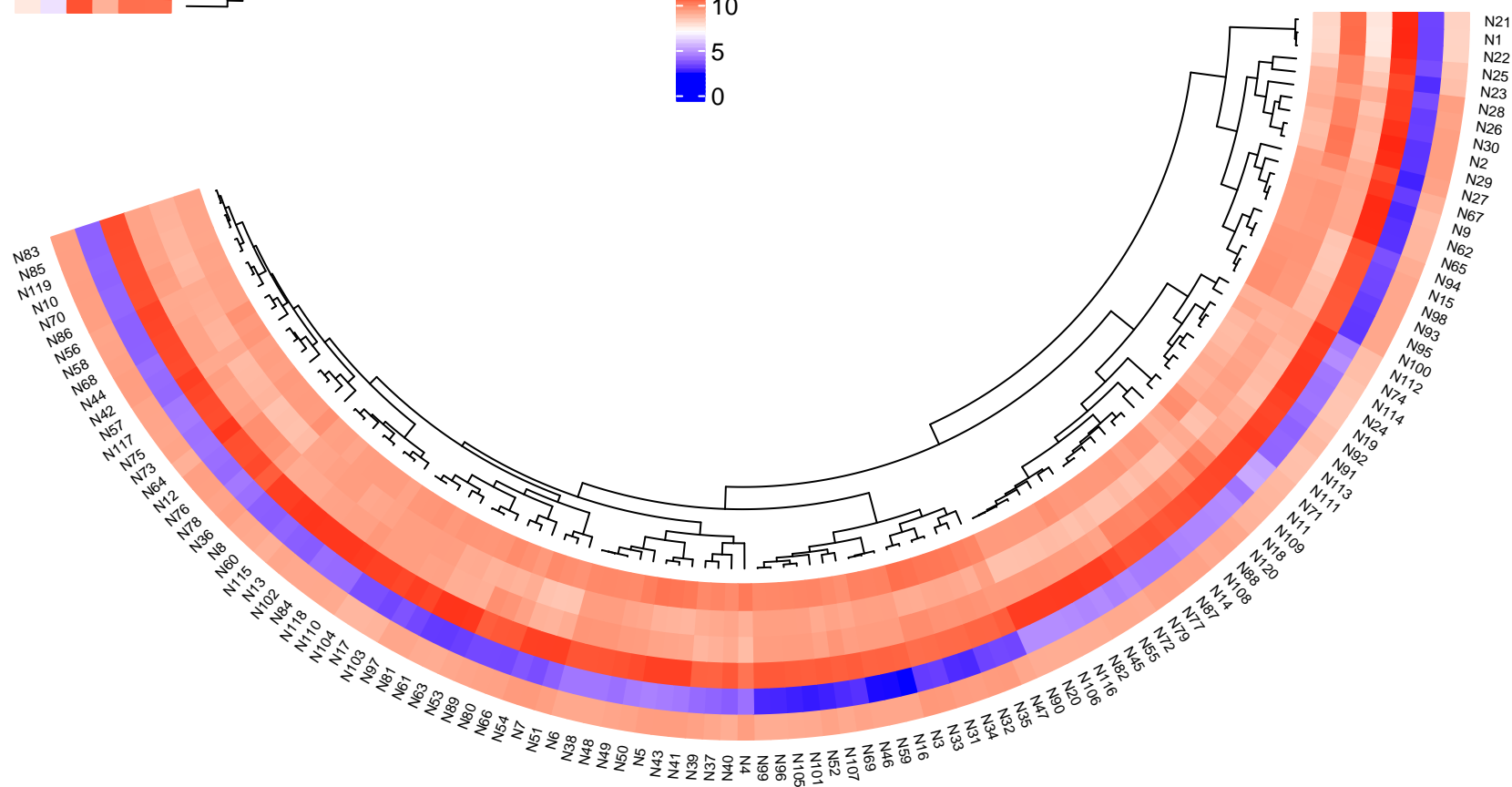

Supplement: Supplementary 2 — Figure S2: using synthetic minority oversampling technique data, differential expression levels of six genes between the healthy group (the bottom fan-shaped region) and IPF group (the top fan-shaped region). [file 1685384.f2.pdf]

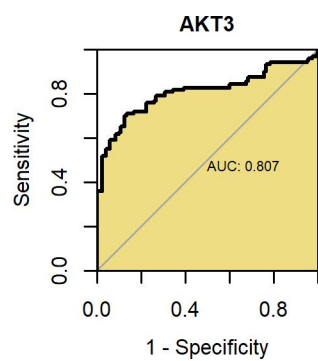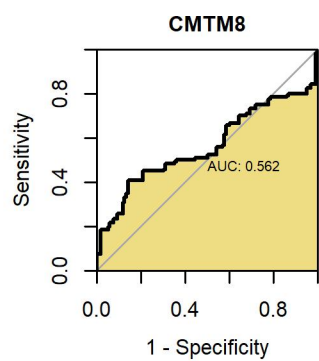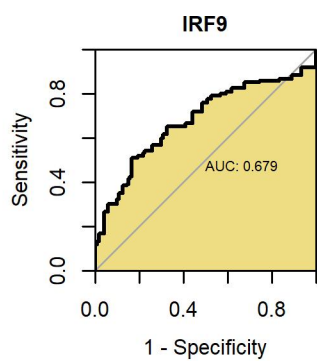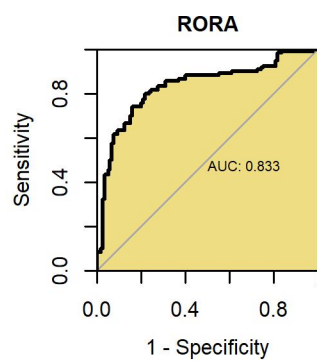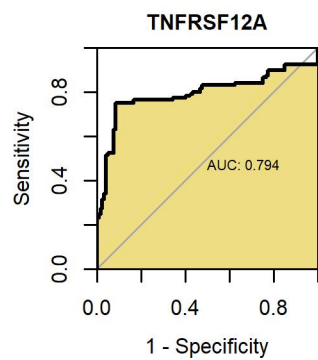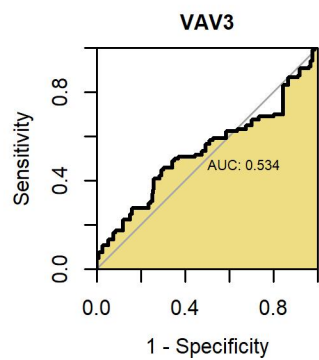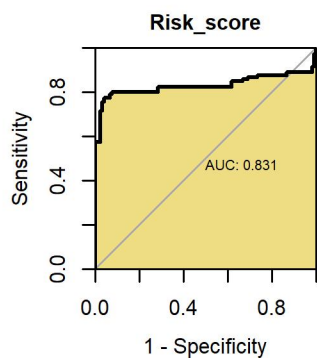

Supplement: Supplementary 3 — Figure S3: using synthetic minority oversampling technique data, the accuracy of m5CPS and its constitutive genes in distinguishing IPF bronchoalveolar lavage from healthy lung bronchoalveolar lavage. AUC: area under the curve. [file 1685384.f3.pdf]
